# Harnessing haploid‐inducer mediated genome editing for accelerated maize variety development

**DOI:** 10.1111/pbi.14608

**Published:** 2025-02-12

**Authors:** Lina Li, Xiao Fu, Xiantao Qi, Bing Xiao, Changling Liu, Qingyu Wu, Jinjie Zhu, Chuanxiao Xie

**Affiliations:** ^1^ State Key Laboratory of Crop Gene Resources and Breeding, Institute of Crop Sciences Chinese Academy of Agricultural Sciences Beijing China; ^2^ National Nanfan Research Institute (Sanya) Chinese Academy of Agricultural Sciences Sanya Hainan China; ^3^ State Key Laboratory of Efficient Utilization of Arid and Semi‐Arid Arable Land in Northern China, Institute of Agricultural Resources and Regional Planning Chinese Academy of Agricultural Sciences Beijing China

**Keywords:** genome editing, haploid induction, HI‐Edit/IMGE, maize, seed industrial application

## Abstract

The integration of haploid induction and genome editing, termed HI‐Edit/IMGE, is a promising tool for generating targeted mutations for crop breeding. However, the technical components and stacking suitable for the maize seed industry have yet to be fully characterised and tested. Here, we developed and assessed three HI‐Edit/IMGE maize lines: Edit^*Wx*^, Edit^*Sh*^, and Edit^*Wx*&*Sh*^, using the haploid inducer CHOI3 and lines engineered using the CRISPR‐Cas9 system targeting the *Waxy1* (*Wx1*) and *Shrunken2* (*Sh2*) genes. We meticulously characterised the HI‐Edit/IMGE systems, focusing on copy numbers and the mutant alleles *mtl* and *dmp*, which facilitate haploid induction. Using B73 and six other parental lines of major commercial varieties as recipients, HI‐Edit/IMGE demonstrated maternal haploid induction efficiencies ranging from 8.55% to 20.89% and targeted mutation rates between 0.38% and 1.46%. Comprehensive assessment verified the haploid identification, target gene editing accuracy, genome background integrity, and related agronomic traits. Notably, Edit^*Wx*&*Sh*^ successfully combined distinct CRISPR‐Cas9 systems to induce multiple desired mutations, highlighting the potential of HI‐Edit/IMGE in accelerating the integration of edited traits into commercial maize varieties. Our findings underscore the importance of meticulous *Cas9* copy number characterisation and highlight potential challenges related to somatic chimerism. We also validated the performance of single‐cross haploids derived using the HI‐Edit/IMGE process. Our results confirm the industrial applicability of generating targeted mutations through pollination and provide critical insights for further optimising this technology.

## Introduction

The burgeoning field of CRISPR‐Cas‐based genome editing holds immense promise for transforming agricultural practices by enabling precise and efficient gene editing in plants (Chen *et al*., 2019). Central to this groundbreaking technology is the efficient delivery of the CRISPR‐Cas9 system to crop varieties for industrial seed applications. Despite their potential, the transformation and regeneration of genetically modified plants, particularly in recalcitrant species, are often impeded by inefficiencies and genotype‐dependent processes, which are especially prevalent in monocotyledonous crops such as maize. (Chen *et al*., 2022). Traditional alternatives, such as backcross introgression from easier‐to‐transform genotypes, can lead to linkage drag issues. Although using stably expressed CRISPR‐Cas in backcrossing can address some of these concerns, it remains a time‐ and resource‐intensive strategy (Li, Liu, *et al*., 2017). In addition, the BREEDIT pipeline and its integration with haploid induction have shown significant potential for accelerating CRISPR‐Cas‐mediated multiplex gene editing to address complex traits (Impens *et al*., 2023; Lorenzo *et al*., 2023). However, the practical application of these strategies in elite lines remains limited by the challenges associated with genetic transformations in diverse genetic backgrounds.

To overcome these limitations, a novel approach that merges gene editing with haploid induction has been proposed, known as HI‐Edit, in crops such as maize, Arabidopsis, and wheat (Kelliher *et al*., 2019), or IMGE in maize (Wang *et al*., 2019). HI‐Edit/IMGE technology utilises haploid inducer lines containing CRISPR‐Cas gene editors to modify target genes in recipient plants, yielding improved haploid plants. This process accelerates the integration of new gene‐edited traits into recipient varieties through rapid haploid induction followed by a doubled haploid (DH) process, circumventing the need for stable genetic transformation and multiple backcross generations, thus operationalising the concept referred to as “targeted mutations through pollination.”

**Figure 1:**
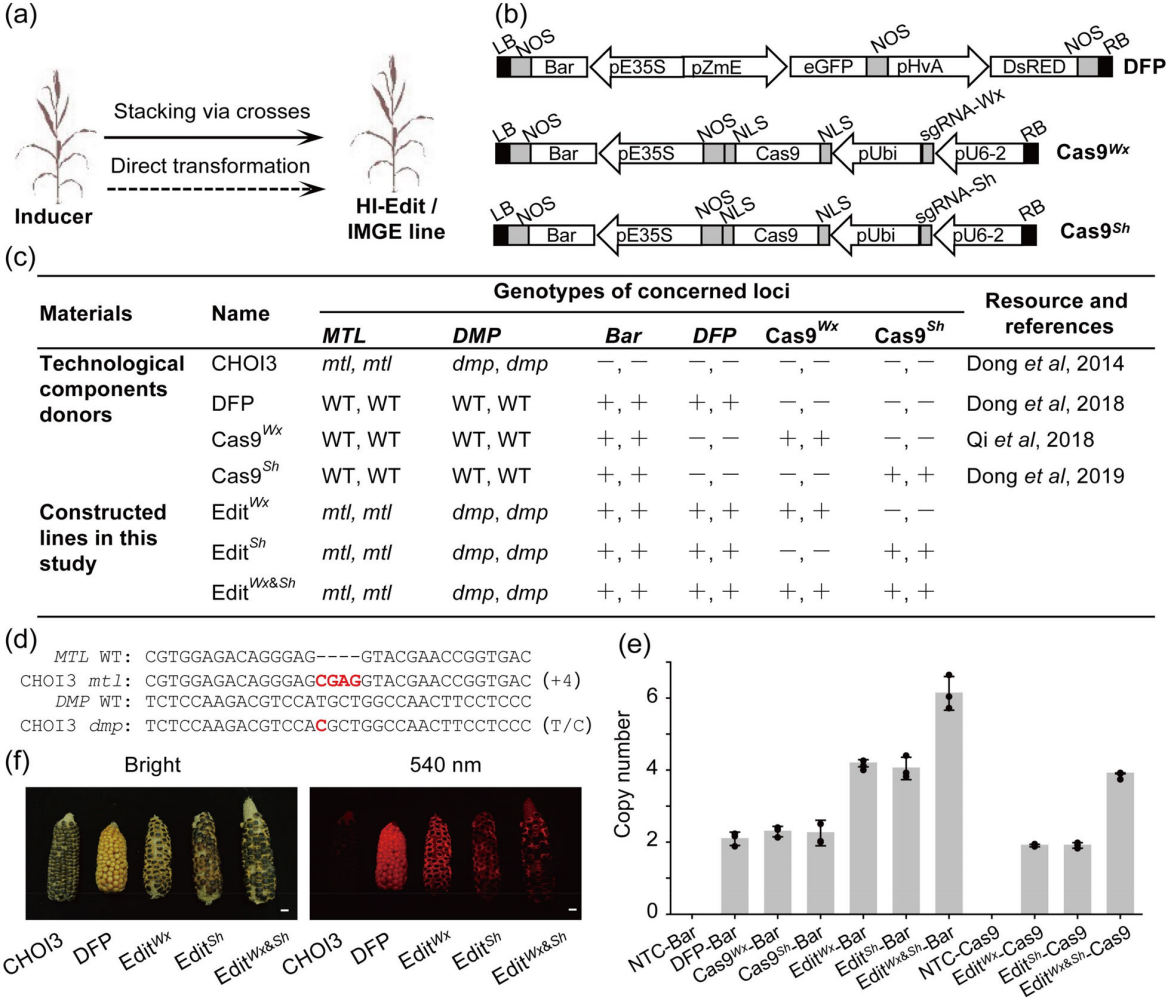
Development of HI‐Edit/IMGE Lines for Efficient Maize Seed Industrial Applications. (a) Overview of the main approaches employing CRISPR‐Cas genome editing. (b) Schematic T‐DNA constructs of double fluorescence protein (DFP) and *WX1* and *SH2* editor (CRISPR‐Cas9 constructs targeting the *ZmWx1* or *ZmSh2* gene). LB, T‐DNA left boarder; Bar, *BlpR* gene; Nos, *NOS* terminator; eGFP and DsRED, driven by the maize embryo‐specific promoter pZmE and barley aleurone‐specific promoter pHvA, respectively; NLS (left), nucleoplasmin; NLS (right), SV40 NLS; Cas9, Cas9; Ubi, ZmUbiquitin promotor; RB, T‐DNA right boarder; pE35S, enhanced *CaMV35S* promoter; U6‐2, ZmU6 Pol III promoter. (c) Description of the plant resources used to stack haploid induction, haploid identification, and gene‐editing parents to develop Edit^*Wx*^, Edit^*Sh*^, and Edit^*Wx*&*Sh*^. (d) Genotyping validation of the *ZmMTL* and *ZmDMP* loci in developed HI‐Edit/IMGE lines. (e) Quantitative analysis of the copy numbers of the Bar and Cas9 genes in the parents and HI‐Edit/IMGE lines. Data represent the mean ± SEM from three independent experiments. (f) Visual representation of the ears of the developed HI‐Edit/IMGE lines (Edit^*Wx*^, Edit^*Sh*^, and Edit^*Wx*&*Sh*^) under both bright and 540 nm excitation light view.

Maize, the largest commercial seed industry crop, is a critical target for the advancement of agricultural technologies. Improvements in genetic modification and breeding techniques can significantly affect global food production. Despite these theoretical advantages, little progress has been made in advancing HI‐Edit/IMGE for seed industry applications, especially for major crops such as maize. The construction of an HI‐Edit/IMGE system can be approached through direct transformation of the inducer or a backcrossing strategy to integrate the necessary components (Figure 1a). Direct transformation, which HI‐Edit/IMGE aims to circumvent, has been successful only recently in maize (Tian *et al*., 2024). Efforts to screen haploid inducer lines with high genetic transformation efficiencies for HI‐Edit/IMGE construction in maize have been reported (Delzer *et al*., 2024). However, direct transformations remain a significant challenge for many researchers. The primary objective of this study was to construct and evaluate HI‐Edit/IMGE technology for its applicability in the maize seed industry using stacking components via a crossing approach. This study systematically assessed the critical components required for an effective HI‐Edit/IMGE system, from the initial stacking of technological elements to the final development of a single‐cross hybrid in maize. Furthermore, it provides valuable insights for further optimising this promising technology, which could streamline crop improvement and breeding processes.

## Results

### Construction of HI‐edit/IMGE lines targeting *ZmWx1* and/or *ZmSh2*

We successfully constructed HI‐Edit/IMGE lines capable of targeting the maize genes *ZmWx1* and/or *ZmSh2* by stacking essential technological components (Figure 1b,c). This process involved integrating two haploid inducer genes (Dong *et al*., 2014), dual fluorescent protein (DFP) as a haploid identification marker (Dong *et al*., 2018), and the CRISPR‐Cas9 system targeting *Waxy1* (*Wx1*) (Qi *et al*., 2018) and/or *Shrunken2* (*Sh2*) (Dong *et al*., 2019) genes through strategically crossing donor lines. Maternal haploid induction was facilitated by incorporating both *MTL* and *DMP* mutant alleles from the CHOI3 inducer line (Dong *et al*., 2014), which were verified in the donor lines and subsequently identified in the newly constructed lines (Figure 1d).

Droplet‐digital PCR (ddPCR) was used to screen for and identify homozygous transgenic agents, including DFP, Cas9 targeting *Wx1* (*Cas9* ^*Wx*^), and/or *Sh2* (*Cas9* ^*Sh*^), as detailed in Figure 1c,e and further elaborated in Figures S1 and S2 and Tables S1 and S2. The constructed Edit^*Wx*^ and Edit^*Sh*^ lines contained four copies of the *Bar* gene and two copies of the *Cas9* gene, attributable to the presence of the *Bar* cassette in the DFP and *Cas9* donor constructs (Figure 1b). The Edit^*Wx*&*Sh*^ line, which included all three components, contained six copies of the *Bar* gene and four copies of the *Cas9* gene. These findings indicated that the constructed lines possessed a single diploid and homozygous genetic pattern for haploid induction (*mtl* and *dmp*) and screening (DFP) and the CRISPR‐Cas9 system for targeted mutagenesis.

The original *R1‐nj* marker from the CHOI3 line (Dong *et al*., 2014) was preserved throughout stacking. The Edit^*Wx*^, Edit^*Sh*^, and Edit^*Wx*&*Sh*^ lines expressed both *R1‐nj* and DFP markers, manifesting purple anthocyanin pigmentation in the kernels (Figure 1f, left panel) and red fluorescence upon exposure to 540 nm excitation light (Figure 1f, right panel).

### Haploid induction and genetic characterisation via HI‐edit/IMGE

**Figure 2:**
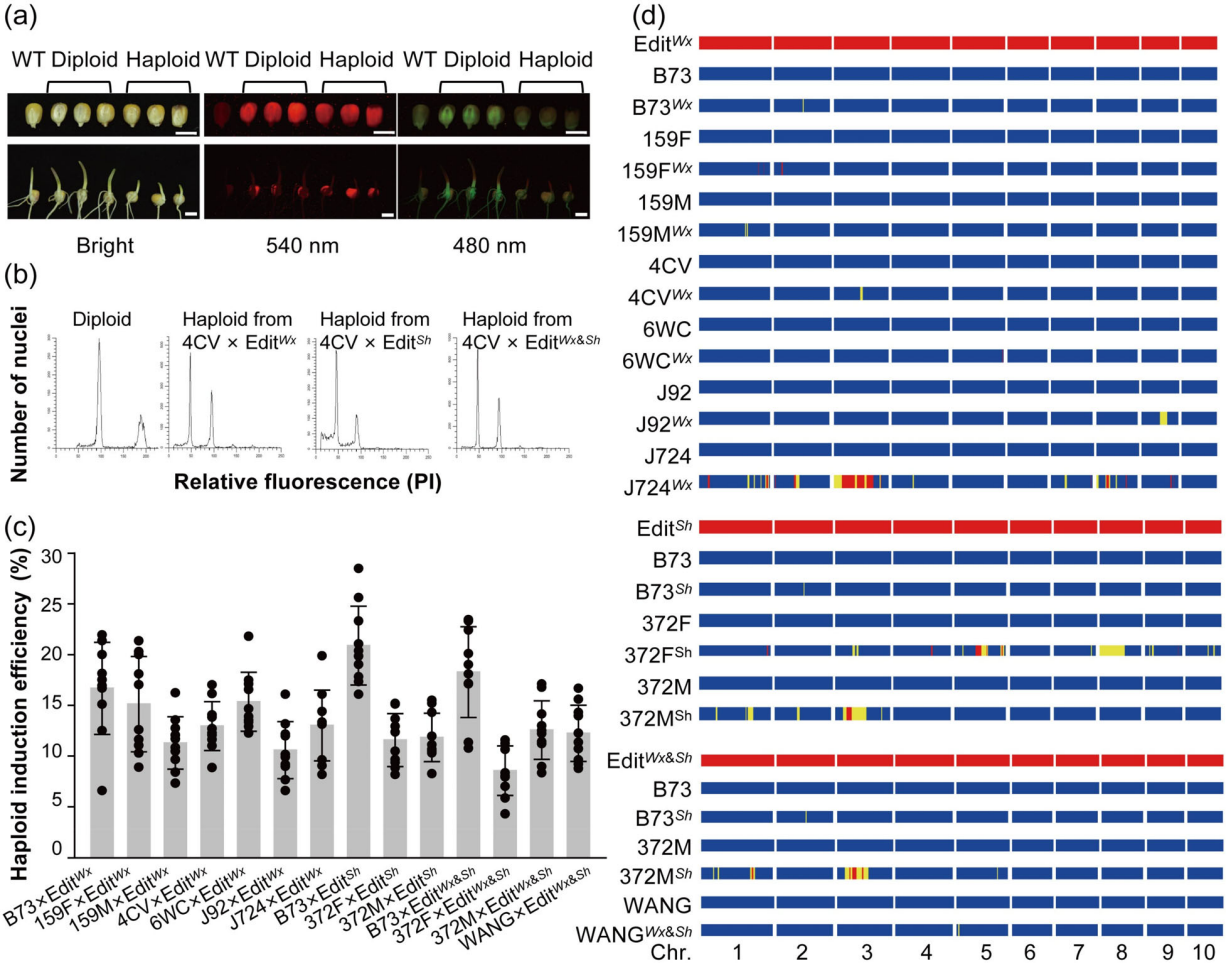
Haploid Induction and Genetic Characterisation via HI‐Edit/IMGE Technology. (a) Discrimination of diploids and haploids in mature seeds and seedlings. Efficient differentiation of diploid and haploid seeds (upper panel) and coleoptiles of seedlings (lower panel) under varying light conditions with the DFP marker. Scale bar = 1 cm. (b) Flow cytometric confirmation of haploid genomic contents. Illustrative flow cytometry profiles validating haploid genome content derived from crosses with Edit^*Wx*^, Edit^*Sh*^, and Edit^*Wx&Sh*^ inducer lines. (c) Haploid induction rates among inbred elite maize. Displayed values represent the mean ± standard deviation (*n* = 10). (d) Genetic background profiling of haploids via the Maize 60 K SNP Array (Thermo Fisher Scientific). The genome profiles were aligned with 10 chromosomes across whole maize genome. Yellow bands indicate a heterozygous pattern, whereas red segmentation indicates the introgression from the inducer in the resulted haploids.

To evaluate the efficacy of haploid induction using Edit^*Wx*^, Edit^*Sh*^, and Edit^*Wx*&*Sh*^, these lines were crossed with a panel of diverse elite inbred parental lines for maternal haploid induction. We identified the haploid progenies using a DFP‐based screening method previously established in our laboratory (Dong *et al*., 2018). This method relies on differential fluorescence emitted by the embryo and aleurone cells of seeds (Figure 2a, upper panel) or young seedlings (Figure 2a, lower panel). Haploid seeds and seedlings were distinguished by the absence of enhanced green fluorescent protein (eGFP)‐induced green fluorescence and the presence of DsRED‐induced red fluorescence. In young seedlings, the absence of green fluorescence in the hypocotyl post‐germination was also a clear indicator of haploidy (Figure 2a, lower panel, Figure S3b). Our observations suggest that DFP offers greater stability and reliability than *R1‐nj* (Figure S3a). DFP detection demonstrated the capability to identify markers within the first few days post‐germination, offering an effective alternative for haploid selection (Figure S3b). This method extended the viable haploid screening timeframe, enhancing the selection process.

The haploid status of the putative haploid offspring was further confirmed by flow cytometry, a technique that accurately measures genomic DNA content, ensuring the precise identification of haploid individuals (Figure 2b). We quantified haploid induction rates (HIRs) across different inbred lines (Figure 2c). The Edit^*Wx*^ inducer line demonstrated an HIR ranging from 10.58% to 16.67% when crossed with seven elite inbred lines. In contrast, the Edit^*Sh*^ inducer line showed a slightly higher HIR, with values ranging from 11.57% to 20.89% across three elite inbred lines. The combined Edit^*Wx*&*Sh*^ inducer lines exhibited an HIR ranging from 8.55% to 18.27% across four elite inbred lines.

To elucidate the genetic background of the induced haploids, we employed the Maize 60 K SNP Array, a high‐throughput genotyping platform, to provide a detailed profile of their genome composition (Figure 2d). Our analysis aimed to confirm the purity and uniformity of the haploid genome across 10 chromosomes of the maize genome. Most haploid individuals exhibited consistent genomic compositions across all 10 chromosomes, matching those of their respective initial lines. This uniformity is indicative of the successful induction of maternal haploids using HI‐Edit/IMGE technology, aligning with the goal of generating genetically pure haploid lines for breeding. However, a subset of haploid individuals displayed a “heterozygous” genome composition, which paradoxically suggests the presence of both inducer and recipient genomes within these regions, as highlighted in yellow in Figure 2d. This unexpected observation was attributed to somatic chimerism in young haploid seedlings, where some chimeric cells contained fragmented inducer DNA owing to an incomplete chromosome elimination process. Moreover, we observed a few significant introgressions of genomic segments from the inducer line in haploid genomes, as highlighted in red in Figure 2d. For instance, in J724^*Wx*^, we detected large red segments on chromosome 3, suggesting that the incompletely eliminated segments from the inducer line underwent recombination and replacement in the haploid line. This rare introgression event likely resulted in the induction of permanently preserved genomic fragments in the constructed lines.

### Targeted mutagenesis efficiency in elite parental lines via HI‐edit/IMGE

**Figure 3:**
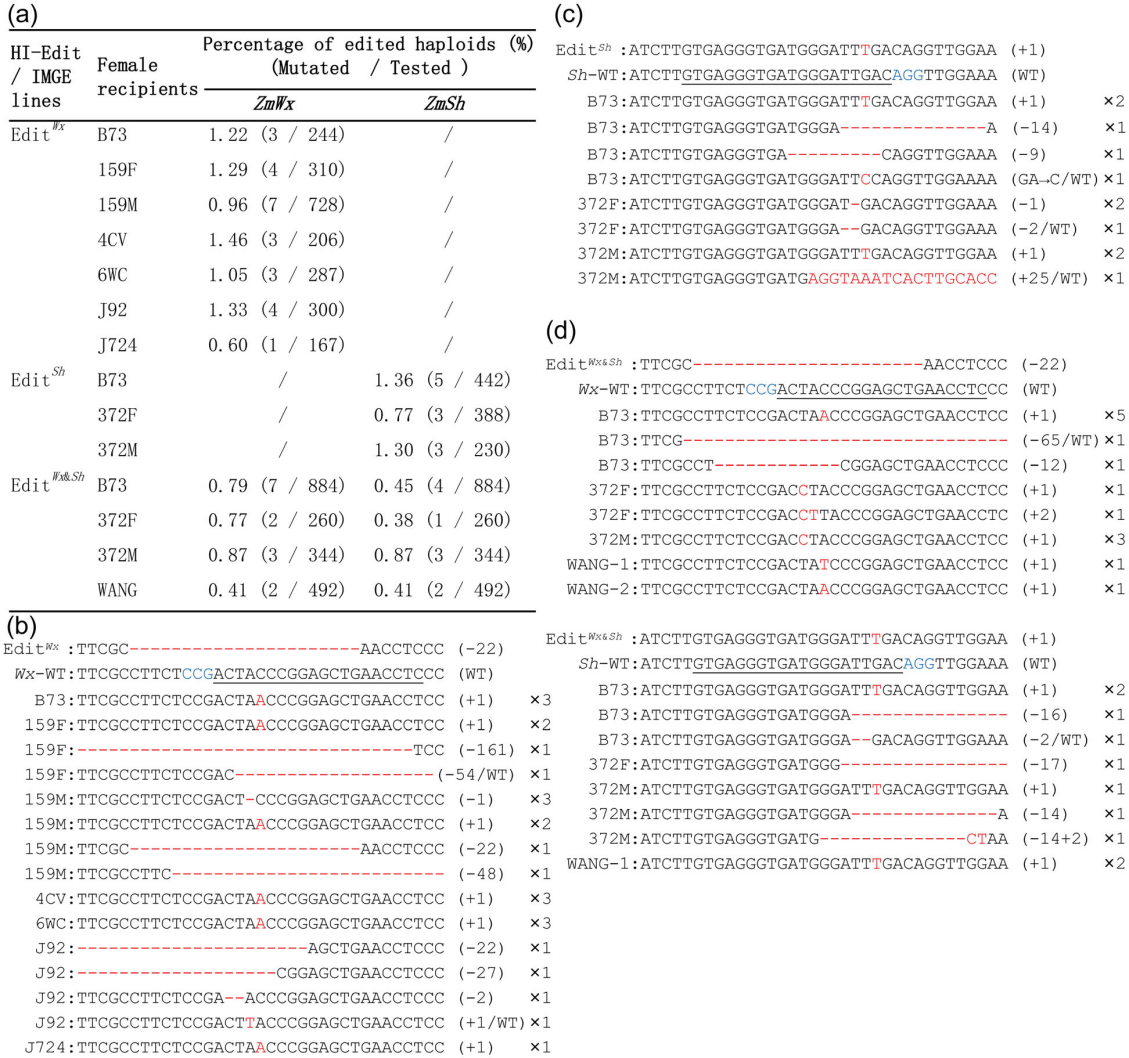
Targeted mutagenesis in elite maize lines using developed HI‐Edit/IMGE lines. (a) Haploid editing efficiency across elite maize varieties. Comparison of targeted mutation efficiency in various elite maize backgrounds via the application of Edit^*Wx*^, Edit^*Sh*^, and Edit^*Wx*&*Sh*^ systems. (b) Alignment depicting the patterns of targeted mutations at the *ZmWx1* locus in haploid plants generated via Edit^*Wx*^. The mutation DNA is shown in red. The mutation type and individual number are listed on the right of each alignment. The underlined sequences indicate the single‐guide RNA association region of CRISPR‐Cas9. (c) Sequence alignments illustrating the precise edits introduced at *ZmSh2* target sites in haploids following the use of Edit^*Sh*^. (d) Single and dual targeting efficiency of Edit^*Wx*&*Sh*^. Among them, WANG‐1 concurrently achieves both *ZmWx1* and *ZmSh2*‐targeted mutations.

We crossed Edit^*Wx*^, Edit^*Sh*^, Edit^*Wx*&*Sh*^, and distinct inbred maize lines, generating 2242, 1060, and 1980 haploids, respectively (Figure 3a). Pooled screening followed by duplicate Sanger sequencing identified 25 individual haploids with target mutations in the *ZmWx1* gene (Figure 3b). Editing efficiency for *ZmWx1* varied among haploids from different inbred lines, ranging from 0.60% to 1.46%. The most common mutations were +1 base‐pair insertions, typical of CRISPR‐Cas9 activity, and deletions near the Cas9 cleavage site, with the largest deletion being −161 base pairs. Furthermore, the two haploid lines with *ZmWx1* target site genotypes were −54/WT and +1/WT (Figure 3b).

For *ZmSh2*, 11 haploids harboured mutations at the target site (Figure 3c). The editing efficiency for *ZmSh2* was between 0.77% and 1.36%, with mutations primarily consisting of +1 base‐pair insertions and minor deletions. Additionally, the three haploid lines with *ZmSh* target site genotypes were identified as −2/WT, +25/WT, and GA → C/WT. For the Edit^*Wx*&*Sh*^ lines (Figure 3d), we explored the possibility of inducing double mutations in both *ZmWx1* and *ZmSh2*. Genotypic analysis showed targeted editing efficiencies for *ZmWx1* between 0.41% and 0.87% and for *ZmSh2* between 0.38% and 0.87%. Similarly, haploid genotype analysis revealed the presence of a −65/WT *Wx1* mutation and a −2/WT *sh2* mutation. Notably, only one haploid individual, Wang‐1, displayed simultaneous editing at both the *ZmWx1* and *ZmSh2* loci, indicating that dual‐target editing with HI‐Edit/IMGE is feasible with a sufficiently large haploid population.

In this study, we critically assessed the efficiency of a targeted mutagenesis system. It is essential to acknowledge that our evaluation may have underestimated the true efficiency, and a few large fragment deletions could not be detected because the primers were designed to score the amplification regions. Additionally, a small number of unexpected heterozygous target genotypes were identified in the haploids produced by Edit^*Wx*^, Edit^*Sh*^, and Edit^*Wx*&*Sh*^ (Figure 3b–d), suggesting the presence of a few chimeric individuals. This finding is corroborated by the results shown in Figure 2d, which indicate that the potential for incomplete chromosome elimination (Li, Liu, *et al*., 2017) from the inducer genome must be considered during haploid induction. These insights will provide valuable guidance for future research in this field.

### Phenotypic validation of target edits at parental and hybrid levels

**Figure 4:**
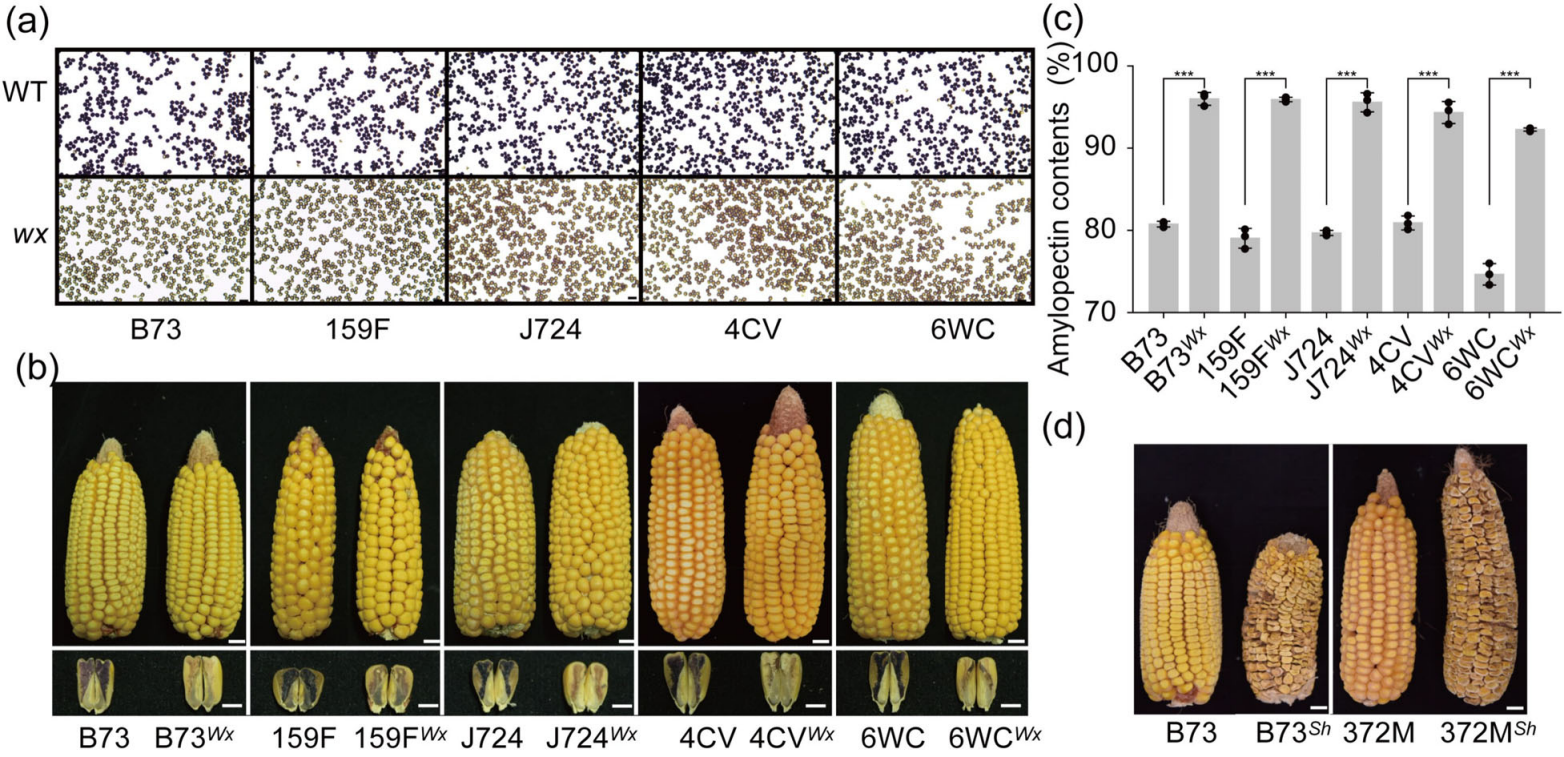
Typical phenotypes of the *ZmWx1* and *ZmSh2* edited inbred lines. (a) Mature pollens of wild‐type inbreds and wx‐edited after KI/I_2_ staining. Scale bar = 100 μm. (b) Comparison of harvested ear and the longitudinal section of kernel after KI/I_2_ staining between wild‐type maize and edited mutant. Scale bar = 1 cm (for ears). Scale bar = 0.5 cm (for kernels). (c) Amylopectin content (%) in seed starch. Data are presented as the mean ± SEM (*n* = 3). ***P < 0.001 indicates significant differences among the *ZmWx*‐edited inbreds and the wild‐type control under the two‐tailed Student's *t*‐test. (d) Typical ears after *ZmSh2* were edited using HI‐Edit/IMGE. Scale bar = 1 cm.

**Figure 5:**
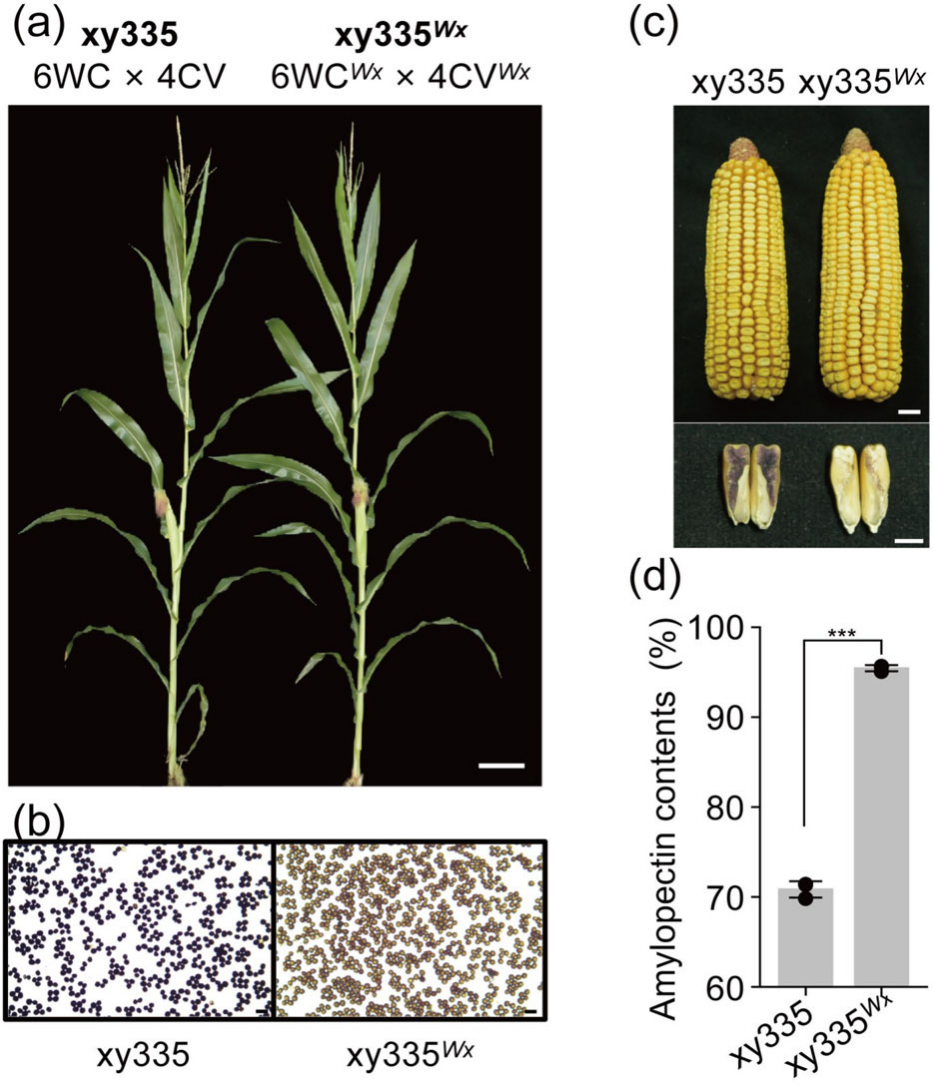
Phenotypic validation of the created single‐cross hybrid using the HI‐Edit/IMGE approach. (a) Appearance comparison of *ZmWx*‐edited hybrid and wild‐type hybrids. A typical single‐cross hybrid of xy335 (6WC × 4CV) was tested. Photos displayed were captured after sowing for 57 days. (b) Mature pollens of *Wx*‐edited and wild‐type hybrid after KI/I_2_ staining. Scale bar = 100 μm. (c) Comparison of harvested ear and the longitudinal section of kernel after KI/I_2_ staining between wild‐type maize and edited mutant hybrid. Scale bar = 1 cm. (d) Amylopectin content (%) in seed starch. Data are presented as the mean ± SEM (*n* = 3). ***P < 0.001 indicates significant differences of xy335^*Wx*^ from the wild‐type xy335 under the two‐tailed Student's *t*‐test.

To phenotypically validate the effects of the targeted edits induced by HI‐Edit/IMGE, we selected representative parental lines (Figure 4) and a single‐cross hybrid (Figure 5) for phenotypic assessment. Haploids generated from Edit^*Wx*^ underwent chromosome doubling to produce DH mutant lines designated as B73^*Wx*^, 159F^*Wx*^, J724^*Wx*^, 4CV^*Wx*^, and 6WC^*Wx*^. In addition, two DH mutant lines for *ZmSh2*, B73^*Sh*^ and 372M^*Sh*^, were successfully developed.

Iodine staining of pollen (Figure 4a) and examination of the kernel endosperm from longitudinally sectioned kernels (Figure 4b, lower panels) revealed typical phenotypic changes associated with the *wx1* mutation. The endosperm starch content and amylopectin levels were measured and confirmed (Figure 4c). A comparison of grain yield between the mutant lines and their wild‐type counterparts showed significant differences (Table S3), whereas ear size remained largely unaffected. However, kernel appearance was altered due to endosperm starch composition changes (Figure 4b, upper panels). These findings are consistent with those of our previous study using the CRISPR‐Cas9 system (Dong *et al*., 2019; Qi *et al*., 2018, 2020a). For the *ZmSh2* DH mutant lines, the characteristic shrunken kernel phenotype was evident in the mature ears (Figure 4d), as observed in our previous CRISPR‐Cas9‐generated knockout mutants (Dong *et al*., 2019).

Crossing 4CV^*Wx*^ with 6WC^*Wx*^ yielded a single‐cross mutant hybrid, xy335^*Wx*^, which was created via HI‐Edit/IMGE for phenotypic validation. Iodine staining of pollen (Figure 5b), endosperm starch (Figure 5c), and amylopectin content were assessed, revealing that xy335^*Wx*^ had a 95.45% amylopectin content, a significant increase from that of the wild‐type (70.83%) (Figure 5d). Field images of the mutant and wild type hybrids at the flowering stage (Figure 5a) showed no significant differences in plant appearance or phenotype.

## Discussion

The transition from theory to practice underscores the comprehensive and mature state of genome editing and highlights its importance in modern agriculture. Genome editing technologies have matured to enable precise mutation targeting, from single‐nucleotide edits to large genomic rearrangements. The current suite of tools, including CRISPR‐Cas systems as well as base and prime editing, allows for the specificity and control of genetic modifications (Li *et al*., 2024). With these advancements, the focus has shifted to applications, particularly to the efficient delivery of CRISPR‐Cas systems to crop varieties in the seed industry. Delivery technology is crucial, as it ensures that the gene‐editing components reach the target cells effectively, facilitating trait enhancement in crops.

### Landscape of current efforts on genome editing deliveries

Genome editing delivery is characterised by various innovative strategies, each designed to enhance the precision and efficiency of genetic modifications in plants. These methods include *Agrobacterium*‐mediated gene transfer (Hamilton *et al*., 1996; Yang *et al*., 2024), particle bombardment for direct DNA delivery (O'Kennedy *et al*., 2011), and virus‐mediated delivery (Oh *et al*., 2021). The induction of meristems (Debernardi *et al*., 2020; Yang *et al*., 2024) and graft mobility techniques (Yang *et al*., 2023; Zhang *et al*., 2016) leverage the natural processes of plants for efficient editing. Protoplast transfection (Woo *et al*., 2015) and cut‐dip‐budding (Cao *et al*., 2023) systems provide culture‐free genetic modification methods, whereas nanoparticle (Kwak *et al*., 2019; Wang *et al*., 2022) and cell‐penetrating peptide (Patel *et al*., 2019) technologies push the boundaries of delivery innovation. Together, these approaches aim to overcome existing challenges and streamline crop improvement.

### Harnessing HI‐edit/IMGE: Overcoming limitations of traditional gene‐editing deliveries

The advent of HI‐Edit/IMGE technology represents a paradigm shift in the application of gene editing to maize, offering distinct advantages over conventional delivery methods. Unlike established yet often cumbersome *Agrobacterium*‐mediated tissue culture transformations (Hamilton *et al*., 1996; Yang *et al*., 2024), HI‐Edit/IMGE provides a more accessible and efficient alternative. While mature, traditional methods are encumbered by their reliance on laborious tissue culture procedures and are frequently stymied by the specific genotypes of recipients, thereby limiting their applicability across diverse genetic backgrounds (Bekalu *et al*., 2023). In contrast, integrating haploid induction with gene editing technology through HI‐Edit/IMGE circumvents these limitations. This innovative approach enables the unencumbered delivery of gene editing components, streamlining the process to a level that is both rapid and straightforward. HI‐Edit/IMGE facilitates targeted improvements in any material, aligning with modern breeding objectives of precision and efficiency. By achieving “targeted mutations through pollination,” HI‐Edit/IMGE not only enhances the speed of genetic enhancement but also broadens the scope of genetic modification, making it an invaluable tool for maize breeders (Dong *et al*., 2014; Kelliher *et al*., 2019; Wang *et al*., 2019) and potentially other crops (Budhagatapalli *et al*., 2020; Kelliher *et al*., 2019; Li *et al*., 2021).

### Present challenges and paths to optimisation of HI‐edit/IMGE

Despite its potential, the application of HI‐Edit/IMGE technology faces several challenges. The editing process is constrained by a narrow temporal window, necessitating precise intervention post‐fertilisation and before paternal chromosome elimination (Zhao *et al*., 2013). This brief period of biological activity of the gene editing machinery often leads to low haploid editing efficiency.

Several strategic optimisations can be implemented to enhance the efficiency of the HI‐Edit/IMGE system. First, increasing the frequency of active edits at the target site is essential to overcome the limitations imposed by short active temporal windows. For gene knockout applications, it is advisable to design and screen a diverse array of target sites and subsequently select the most effective ones for further optimisation (Chuai *et al*., 2017). Second, the deployment of ‘super‐promoter’ elements, such as CmYLCV, which is known for its robust activity (Stavolone *et al*., 2003), can significantly boost editing efficiency. Additionally, promoters preferred during the sperm‐to‐zygote transition phase may be exploited to enhance the temporal specificity of gene editing. Third, chromatin accessibility positively correlates with the success rate of Cas9‐mediated gene editing. Co‐expression of transcriptional activators, demethylases, and other factors that enhance chromatin accessibility at the target site may substantially improve editing efficiency (Chen *et al*., 2016; Chung *et al*., 2020). However, empirically validating these strategies to confirm their efficacy in the context of haploid induction and gene editing systems is crucial. This validation is necessary to ensure that the proposed enhancements translate into tangible improvements in gene editing outcomes.

For multiple‐target site editing, we developed Edit^*Wx*&*Sh*^, which can edit two target genes simultaneously. In our experiments, we successfully identified a haploid plant with concurrent edits in both genes, although the observed efficiency was exceedingly low at 0.05% (1/1980). The results demonstrate the feasibility of the HI‐Edit approach in achieving targeted edits within the elite maize germplasm and underscore the critical need for larger induced populations to improve the likelihood of recovering multiple edits simultaneously. Lorenzo *et al*. (2023) effectively employed the BREEDIT pipeline to systematically target multiple genes, facilitating the exploration of gene families and complex trait interactions. Similarly, Impens *et al*. (2023) combined multiplex gene editing with haploid induction, enabling the rapid generation of multiplex mutants to study complex genetic networks. Despite these advances, the production of multisite editing outcomes depends on the stable and sustained expression of the CRISPR‐Cas system. HI‐Edit prioritises simplicity, speed, and direct applicability to elite commercial breeding lines, making it suitable for practical breeding. Future efforts should aim to integrate systematic multiplexing strategies with the streamlined HI‐Edit process to maximise efficiency in both research and breeding contexts. We acknowledge that future advancements in improving single‐target editing efficiency will provide a foundation for further developing multi‐target editing systems that hold significant application potential. Nevertheless, our study provided a comprehensive assessment of HI‐Edit/IMGE viability in maize, offering critical insights and a solid foundation for future technological refinements. While acknowledging these ongoing challenges, our study maps a trajectory for their resolution, promising significant progress in the application of HI‐Edit/IMGE in agricultural biotechnology.

### The importance of tracing active CRISPR‐Cas copies during HI‐edit/IMGE development

Our research underscores the pivotal role of accurate copy number characterisation in HI‐Edit/IMGE systems. Among the 11 HI‐Edit/IMGE lines constructed in parallel, only the 3 lines characterised by the explicit identification of their component copy numbers, which were the subjects of this study, were successful. This highlights the necessity for meticulously identifying component copy numbers, which is essential for successfully developing advanced genetic technologies. A significant insight from our study was the critical importance of identifying a single active copy of a homozygous CRISPR‐Cas line within various donor families at the outset. Tracking this specific copy throughout the HI‐Edit/IMGE line construction process is crucial to achieving successful outcomes. The reason for this strategy is rooted in the observation that although transformants may possess multiple CRISPR‐Cas copies, not all are equally active. Identifying active copies at later stages can lead to failure, emphasising the importance of early and precise tracking of copies with verified activity. In addition, multiple copy transgenes may lead to epigenetic silencing, a phenomenon that occurs when an increase in the transgenic construct copy number triggers regulatory mechanisms that suppress gene expression (Assaad *et al*., 1993).

### HI‐edit/IMGE mediated genome introgression and somatic chimerism in haploids

Previous research has demonstrated that the fragmentation of sperm chromosomes during pollen mitosis directly causes paternal chromosome elimination and post‐fertilisation haploid induction (Li, Meng, *et al*., 2017). In maize, double fertilization occurs between 14 and 28 h after pollination (HAP), with the first mitotic event occurring between 26 and 36 HAP (Wu *et al*., 2011; Zhou *et al*., 2017). Typically, within 1 week following pollination, most paternal DNA is eliminated, but some fragments persist until 12 days (7.37%) to 15 days (2.38%) post‐pollination (Qiu *et al*., 2014; Zhao *et al*., 2013).

Genomic introgression and/or somatic mosaicism were attributed to pollination from the inducer line. During this phase, it is plausible, albeit rare, for paternal chromosomal fragments to stably integrate into the maternal haploid genome, forming introgression segments that have the potential for heritability (Amundson *et al*., 2021; Zhao *et al*., 2013). This is consistent with the findings of the current study, which detected haploid genotypes at specific loci that demonstrated introgression from the inducer, as indicated in red in Figure 2d. Furthermore, the emergence of aneuploidy in developing seedlings resulting from a partial failure to eliminate the inducer chromosomes can precipitate somatic chimerism. This is indicated by the yellow colour in Figure 2d. It is crucial to emphasise that most somatic chimerism events in haploid individuals are not expected to pass on to subsequent generations. Our findings confirm that the HI‐Edit/IMGE method is an effective tool for generating maternal haploids, underscoring the need for further research on genomic introgression and somatic chimerism mechanisms to enhance this technique and maintain the genetic purity of haploid lines.

## Experimental procedures

### Plant materials and growth conditions

The original haploid inducer line, CHOI3 (Dong *et al*., 2014), was kindly provided by Professor Shaojiang Chen from the China Agricultural University. We utilised the haploid detection line DFP (Dong *et al*., 2018) and the gene‐editing lines Cas9^*Wx*^ (Qi *et al*., 2018) and Cas9^*Sh*^ (Dong *et al*., 2019), which our group previously developed. Details of the off‐target analysis for both *ZmWx1* and *ZmSh2* targets are provided in Table S5. The DFP line is characterised as a stable, homozygous line with a single‐copy insertion, expressing eGFP specifically in embryos and DsRED in endosperms. Cas9^*Wx*^ and Cas9^*Sh*^ denote homozygous single‐copy lines harbouring stably expressed Cas9‐guided RNA constructs designed to target *ZmWx1* and *ZmSh2* genes, respectively.

Homozygous transgenic lines containing DFP were intercrossed with the CHOI3 line. The produced F1 progenies were crossed with homozygous transgenic lines harbouring CRISPR‐Cas9^*Wx*^ or CRISPR‐Cas9^*Sh*^, followed by at least two selfing generations. Marker‐assisted selection was performed for *MTL*, *DMP*, DFP, *Cas9* ^*Wx*^, and *Cas9* ^*Sh*^. The lines harbouring the homozygous *mtl‐dmp* mutant, as well as DFP, *Cas9* ^*Wx*^, and *Cas9* ^*Sh*^ single‐copy numbers and homozygosity, were selected as haploid inducers with targeted gene‐editing capabilities, named Edit^*Wx*^ and Edit^*Sh*^. Edit^*Wx*^ and Edit^*Sh*^ were intercrossed and self‐pollinated to generate Edit^*Wx*&*Sh*^. The haploid inducer and elite female lines were cultivated in an open field during summer. All test crosses were conducted at a well‐controlled experimental base at the Chinese Academy of Agricultural Sciences in Beijing, China.

### Copy number determination via ddPCR

The copy numbers of the transgene cassettes, *Bar* and *Cas9*, were ascertained using a multiplex quantitative ddPCR approach, as previously described (Qi *et al*., 2020a,b; Zhou *et al*., 2018). Primers and probes for amplifying the *Bar*, *Cas9*, and the endogenous reference gene *ZmADH1* were designed utilising Primer3Plus software (Rozen and Skaletsky, 2000), with details provided in Table S6. Customised fluorescent probes labelled with FAM for *Bar* and *Cas9* and HEX for *ZmADH1* were synthesised. The ddPCR reaction mixture comprised 0.9 μM each of forward and reverse primers, 0.227 μM of probes, 50–100 ng of genomic DNA, and 2× ddPCR Supermix (Bio‐Rad Laboratories, Hercules, CA). Thermal cycling conditions were as follows: an initial incubation at 50 °C for 2 min, denaturation at 95 °C for 10 min, followed by 40 cycles of 95 °C for 15 s for denaturation, and 61 °C for 1 min for annealing and extension (at a ramp rate of 2.5 °C/s). Cycling was concluded with a final extension at 98 °C for 10 min, after which the samples were held at 4 °C. Post‐thermal cycling, the PCR samples were loaded onto a QX200™ Droplet Reader (Bio‐Rad Laboratories) for droplet analysis. QuantaSoft analytical software package v1.6.6.0320 (Bio‐Rad Laboratories) was used to analyse the FAM and HEX fluorescence signals from the droplets. The copy numbers of the target genes were determined by setting thresholds to differentiate between positive and negative droplets, thereby quantifying the precise copy numbers of the *Bar* and *Cas9* transgenes in the samples.

### Haploid identification and mutation detection

The developed lines, Edit^*Wx*^, Edit^*Sh*^, and Edit^*Wx*&*Sh*^, were identified using a DFP marker, which facilitates the specific expression of eGFP in embryos and DsRED in the endosperm, as we previously reported (Dong *et al*., 2018; Qi *et al*., 2020b). Briefly, using a LUYOR‐3425RG fluorescent flashlight (LUYOR, Irvine, CA), we observed green fluorescence in embryos at 480 nm excitation and red fluorescence in endosperms at 540 nm excitation, indicating the presence of DFP markers. F1 seeds derived from crosses with elite maternal and inducer lines were screened for haploidy based on their fluorescence properties. Haploid seeds display colourless embryos and red aleurones under excitation, in contrast to the distinct characteristics of diploid seeds. The eGFP expression in the embryo was monitored throughout germination and was consistently present in the hypocotyl region until the first cotyledon emerged, thereby confirming haploidy. The coleoptile tips of haploids were cut off and immersed in 0.06% colchicine solution to promote chromosomal doubling and then transplanted into nursery containers for further genetic analysis. At the V2 growth stage, leaf samples were collected from four D0 plants for DNA extraction. The DNA was subjected to target amplification, followed by Sanger sequencing to determine the presence of mutations in the genes of interest. To enhance the accuracy of mutation detection, we employed a pooled screening approach in which multiple samples were grouped and collectively sequenced. This method allowed the efficient identification of heterozygous mutations within pools. Once detected, individual samples within the pool were further analysed to confirm the presence of targeted mutations. Haploids containing the desired mutations were transplanted to the field for pollination, resulting in D1 seeds. These seeds were subsequently used for phenotypic analysis to assess the effects of the induced mutations on plant characteristics.

### Flow cytometry

Flow cytometry analysis was conducted to ascertain the ploidy of the potentially haploid seedlings. Leaf samples at the three‐leaf stage were meticulously dissected using a razor blade in a Petri dish containing nuclei extraction buffer. Subsequently, the extracted nuclei were filtered through an 80‐μM filter and stained with Propidium iodide staining buffer. Ploidy status assessments were performed using a FACSCalibur system (BD Biosciences, Franklin Lakes, NJ), and data analysis was performed using ModFit software (Verity Software House, Topsham, ME).

### Chip hybridisation and genome profile analysis

Genomic DNA was extracted from young seedling leaves using the cetyltrimethylammonium bromide method, a standard protocol for plant DNA isolation. The concentration of the extracted DNA was adjusted to 100 ng per sample and quantified using a NanoDrop 2000 spectrophotometer (Thermo Fisher Scientific, Waltham, MA) to ensure accurate DNA input for downstream applications. For high‐throughput genotyping, the Maize SNP60K DNA BeadChip (Thermo Fisher Scientific), which encompasses 61 224 single‐nucleotide polymorphism (SNP) markers evenly distributed across the maize genome, was used (Tian *et al*., 2021). These markers were hybridised onto chips, and individual samples were genotyped. The genotype data were processed using Affymetrix Genotyping Console™ software, version 4.1 (Affymetrix, Santa Clara, CA), a dedicated tool for generating genotype calls from CEL files produced by the GCOS Affymetrix system. The software's analysis is informed by library files containing essential reference information for accurate SNP calling. For data quality assessment, SNPs with low call rates across all samples were filtered from the dataset. Only those SNPs with a call rate exceeding 95.0% and a data quality call score above 0.85 were retained for further analysis per the established criteria (Roorkiwal *et al*., 2018). This stringent selection process ensured that the resulting genome profiles were robust and reliable for subsequent genetic and breeding studies.

### Pollens and seeds starch staining

Fresh anthers were fixed with Carnoy's fixative (composed of ethyl alcohol and acetic acid in a 1:1 ratio) for 24 h. Subsequently, pollen grains were directly stained with Lugol's solution for 5 min and observed under an optical microscope (MSHOT, Guangzhou, China) (Hunt *et al*., 2013). Starch staining was conducted by longitudinally cutting kernels from the middle of the embryo, followed by staining with Lugol's solution, air‐drying, and image capture.

### Starch composition and content and analysis

Starch and amylose contents in maize kernels were measured using a Megazyme K‐AMYL Kit (Megazyme, Bray, Ireland) following the manufacturer's recommended protocols. Briefly, kernels were processed into a fine powder, from which 20–25 mg was used to determine amylose and amylopectin levels. The analysis commenced with dispersing powdered samples in dimethyl sulfoxide (DMSO), which was preheated to facilitate dissolution. Subsequently, the lipids and proteins were precipitated using ethanol, allowing starch granule isolation. The precipitated starch was redissolved in a mixture of heated DMSO and concanavalin A, a lectin that selectively binds to and precipitates amylopectin. After centrifugation, the amylose‐enriched supernatant was separated from the pellet containing the concanavalin A‐amylopectin complex. Both the amylose‐ and total starch‐containing supernatants were subjected to enzymatic hydrolysis to convert the starch polymers into glucose units. The glucose produced was quantified using a colorimetric assay based on the glucose oxidase/peroxidase method, with the absorbance measured at 510 nm. Amylopectin content was calculated by subtracting the proportion of amylose from the total starch content (as a percentage). Each sample was analysed in triplicate to ensure the accuracy and reproducibility of the results.

### Statistical analyses

Data pertaining to phenotypic traits were analysed to present the mean values accompanied by their standard deviations, ensuring a comprehensive representation of the variability observed within the experimental groups. Each experiment was conducted with a minimum of three biological replicates to ensure the reliability of the results. The number of measurements for each experiment is detailed in the corresponding figure legends. For the statistical evaluation of phenotypic data, we used SPSS software (SPSS Inc., Chicago, IL), leveraging its robust analytical capabilities. A two‐tailed Student's *t*‐test was selected as the statistical method to compare the means of mutant and wild‐type samples. This test is appropriate for evaluating the differences between two groups when the samples are independent. The threshold for statistical significance was set at *P* < 0.05, which is the conventional level used to determine whether observed differences are unlikely to have occurred by chance. Any result with a *P*‐value below this threshold was considered to indicate a statistically significant difference between the groups being compared.

## Conflict of interest

The authors declare no conflicts of interest.

## Author contributions

Chuanxiao Xie and Jinjie Zhu designed and supervised the experiments. Lina Li, Xiao Fu, and Xiantao Qi performed the experiment. Lina Li, Jinjie Zhu, Bing Xiao, Qingyu Wu, Changling Liu, and Chuanxiao Xie performed the data analysis. Jinjie Zhu and Lina Li draughted the manuscript. Chuanxiao Xie revised the manuscript.

## Supporting information


**Figure S1** ddPCR verification of copy numbers in each Hi‐Edit/IMGE component donor and developed lines using *the Bar* gene.
**Figure S2** ddPCR verification of copy numbers of CRISPR/Cas9 in Hi‐Edit/IMGE component donor and developed lines using the *Cas9* gene.
**Figure S3** Haploid identification using DFP.
**Table S1** ddPCR data of *Bar* Copy number determination in this study.
**Table S2** ddPCR data of *Cas9* Copy number analysis in this study.
**Table S3** Comparison of the agronomic traits between *ZmWx1*‐edited inbred and its wild type in the same field trial.
**Table S4** Comparison of the agronomic traits between *ZmWx1*‐edited hybrid and its wild type in the same field trial.
**Table S5** Top 20 genome‐wide off‐targets of *ZmWx1* and *ZmSh2* sgRNA.
**Table S6** The primers and probes used in this study.

## Data Availability

The data that supports the findings of this study are available in the supplementary material of this article.
