# Supplementary material for: Harnessing haploid‐inducer mediated genome editing for accelerated maize variety development
Source: Plant Biotechnol J. 2025 Feb 12;23(5):1604–14. doi: 10.1111/pbi.14608 (PMC12018813; doi:10.1111/pbi.14608)
Supplement: Supplementary file 1 — Figure S1 ddPCR verification of copy numbers in each Hi‐Edit/IMGE component donor and developed lines using the Bar gene. Figure S2 ddPCR verification of copy numbers of CRISPR/Cas9 in Hi‐Edit/IMGE component donor and developed lines using the Cas9 gene. Figure S3 Haploid identification using DFP. Table S1 ddPCR data of Bar Copy number determination in this study. Table S2 ddPCR data of Cas9 Copy number analysis in this study. Table S3 Comparison of the agronomic traits between ZmWx1‐edited inbred and its wild type in the same field trial. Table S4 Comparison of the agronomic traits between ZmWx1‐edited hybrid and its wild type in the same field trial. Table S5 Top 20 genome‐wide off‐targets of ZmWx1 and ZmSh2 sgRNA. Table S6 The primers and probes used in this study. [file PBI-23-1604-s001.docx]

(a)

6000 NTC DFP Cas9*Wx* Cas9*Sh* Edit*Wx* Edit*Sh* Edit*Wx*&*Sh*

4000

2000

12000

9000

6000

3000

0 20000 40000 60000 80000 100000

(b)

6000 3271

4000

2000 10458

876 6000 3633

4000

2896 2000 8817

1436 6000 1776 204

4000

3378 2000 14179 1789

0 3000 6000 9000 12000 *ZmADH1*

0 3000 6000 9000 12000 *ZmADH1*

0 3000 6000 9000 12000 *ZmADH1*

6000 4296

4000

2000 10644

850 6000 3505

4000

1941 2000 10713

593 6000 3349 328

4000

1649 2000 11134 999

0 3000 6000 9000 12000 *ZmADH1*

0 3000 6000 9000 12000 *ZmADH1*

0 3000 6000 9000 12000 *ZmADH1*

**Figure S1** ddPCR verification of copy numbers in each Hi-Edit/IMGE component donors

and developed lines using *Bar* gene. (a) Droplet-digital PCR analysis of the *Bar* gene

using a FAM probe (upper panel) and the endogenous diploid reference gene *ZmADH1*

using a HEX probe (lower panel). (b) Quantification of droplet numbers for each sample.

The *Bar* gene amplitudes are represented by the sum of blue and orange droplets, while

the *ZmADH1* reference amplitudes are represented by the sum of green and orange

droplets. Copy numbers for the *Bar* gene were calculated based on the number of droplets

with *Bar*, *ZmADH1*, *Bar*/*ZmADH1* amplitudes, and blank amplitudes.

(a) NTC Edit*Wx* Edit*Sh* Edit*Wx*&*Sh* 12000

9000

6000

8000

6000

4000

0 10000 20000 30000 40000 (b)

12000 2295 4977 12000 2627 2600 12000 2416 6958

9000 9000 9000

6000 1115 2515

0 4000 6000 8000 *ZmADH1*

6000 3070 2978

0 4000 6000 8000 *ZmADH1*

6000 214 597

0 4000 6000 8000 *ZmADH1*

**Figure S2** ddPCR verification of copy numbers of CRISPR/Cas9 in Hi-Edit/IMGE

component donor and developed lines using *Cas9* gene. (a) Droplet-digital PCR analysis of

the *Cas9* gene using a FAM probe (upper panel) and the endogenous diploid reference

gene *ZmADH1* using a HEX probe (lower panel). (b) For the quantification of droplet

counts per sample, the *Cas9* gene signals are denoted by the combined count of blue and

orange droplets, whereas the *ZmADH1* reference signals are indicated by the combined

count of green and orange droplets. The copy numbers of the *Cas9* gene were determined

by the total number of droplets exhibiting *Cas9*, *ZmADH1*, and their combined presence,

along with the droplets showing no signal.

(a)

4CV × Edit*Wx*

6WC × Edit*Wx*

J724 × Edit*Wx*

Putative Diploid Haploid

Putative Diploid Haploid

Bright 480 nm (b)

5 DAG

7 DAG

9 DAG

Bright 480 nm

**Figure S3** Haploid identification using DFP. (a) Screening for putative haploid seeds using

both R1-nj and eGFP florescence of DFP markers. The white arrows highlight two

instances of pseudo-haploids that were identified by the *R1-nj* anthocyanin marker but

were revealed as diploids upon examination with the DFP marker. This observation

suggests that the DFP marker offers greater stability and reliability in distinguishing haploid

from diploid genotypes. Scale bar = 1 cm. (b) Expression of green fluorescent protein in

the seedling hypocotyl in different days after germination. DFP detection has demonstrated

its capability to identify markers within the first few days post-germination, offering an

effective alternative for the selection of haploids. This method extends the viable timeframe

for haploid screening, thereby enhancing the selection process. Scale bar = 1 cm. DAG,

days after germination

**Table S1** ddPCR data of *Bar* Copy number determination in this study.

**Materials**

**FAM**

**Events Droplet Number a**

**HEX** **FAM+HEX** **Empty** **Total**

**Concentration**

**(copies/μl) b**

**Ratio c Number d *Bar*** ***ZmADH1***

**Average Copy Number**

NTC

DFP

DFP

DFP

Cas9*Wx*

Cas9*Wx*

Cas9*Wx*

Cas9*Sh*

Cas9*Sh*

Cas9*Sh*

Edit*Wx*

Edit*Wx*

Edit*Wx*

Edit*Sh*

Edit*Sh*

Edit*Sh*

Edit*Wx*&*Sh*

Edit*Wx*&*Sh*

Edit*Wx*&*Sh*

11 0 0

4147 3772 876

2357 2492 427

2175 2019 235

5069 4814 1436

2453 2072 274

2884 2506 440

1980 1993 204

2634 2139 368

2187 2172 275

5146 2791 850

5963 3188 1065

5284 2822 1173

4099 2242 593

2443 1389 233

4239 2109 607

3677 1327 328

4910 1970 559

6716 2424 1023

15873 15884 16.4

10458 17501 318

9608 14030 216

13095 17054 161

8817 17264 409

14053 18304 169

10915 15865 236

14179 17948 138

12122 16527 204

11856 15940 174

10644 17731 403

10240 18326 463

6193 13126 606

10713 16460 337

13065 16774 194

8778 14519 406

11134 15810 311

10758 17016 401

7784 15901 646

0 / / /

286 1.114 2.228 2.089

230 0.940 1.880

148 1.080 2.160

385 1.063 2.126 2.290

141 1.200 2.400

202 1.170 2.340

138 1.000 2.000 2.250

163 1.250 2.500

172 1.010 2.020

201 2.000 4.000 4.190

225 2.060 4.120

285 2.130 4.260

172 1.960 3.920 4.047

102 1.910 3.820

185 2.200 4.400

103 3.020 6.040 6.133

140 2.860 5.720

195 3.320 6.640

**Note:** a. For copy number variation ddPCR, the *Bar* target was designed with FAM-probe,

and the *ZmADH1* reference was designed with HEX-probe. b. The event droplet numbers

were calculated for the concentration of each gene by QuantaSoft Software. c. Ratio = Bar

target gene concentration/*ZmADH1* reference gene concentration. d. Copy number = Ratio

* NB. The NB here in this study is 2, which means the copy numbers of *ZmADH1* in maize diploid genome.

**Table S2** ddPCR data of *Cas9* Copy number analysis in this study.

**Materials**

**FAM**

**Events Droplet Number a**

**HEX** **FAM+HEX** **Empty** **Total**

**Concentration**

**(copies/μl) b** **Ratio c**

***Cas9*** ***ZmADH1***

**Copy Average Number d Number**

NTC

Edit*Wx*

Edit*Wx*

Edit*Wx*

Edit*Sh*

Edit*Sh*

Edit*Sh*

Edit*Wx*&*Sh*

Edit*Wx*&*Sh*

Edit*Wx*&*Sh*

0 0 0

7272 7492 4977

10449 10522 9577

10272 10586 7731

5227 5578 2600

3333 3464 899

5439 5481 2586

9374 7555 6958

6585 3769 1475

5649 3439 1853

11933 11933

1115 10902

90 11484

965 14092

3070 11275

7512 13410

3236 11570

214 10185

7946 16825

3137 10372

No Call No Call / / /

1294 1367 0.946 1.892 1.903

2830 2920 0.971 1.942

1536 1637 0.938 1.876

733 803 0.912 1.824 1.905

336 352 0.956 1.912

747 755 0.989 1.978

2980 1593 1.870 3.740 3.910

584 298 1.960 3.920

925 474 1.950 3.900

**Note:** a. For copy number variation ddPCR, the *Cas9* target was designed with FAM-probe,

and the *ZmADH1* reference was designed with HEX-probe. b. The event droplet numbers

were calculated for the concentration of each gene by QuantaSoft Software. c. Ratio =

*Cas9* target gene concentration/ *ZmADH1* reference gene concentration. d. Copy number =

Ratio*NB. The NB here in this study is 2, which means the copy numbers of *ZmADH1* in maize diploid genome.

**Table S3** Comparison of the agronomic traits between *ZmWx1*-edited inbred and its wild type in the same field trial.

**Leaf numbers** **Kenerl phenotypes**

**Inbreds**

**Plant height Ear height**

**(cm)** **(cm)** **Above** **Below** **Length ear ear (mm)**

**Width** **Thickness Hundred-grain (mm) (mm)** **weight (g)**

B73 (n=29)

B73*Wx* (n=23)

159F (n=21)

159F*Wx* (n=25)

J724 (n=28)

J724*Wx* (n=29)

4CV (n=18)

4CV*Wx* (n=15)

6WC (n=17)

6WC*Wx* (n=21)

242.03 ± 8.10

246.22 ± 7.56

207.57 ± 9.19

204.04 ± 8.74

273.93 ± 8.06

272.28 ± 7.10

NS

NS

268.94 ± 7.26

250.10 ± 7.32***

91.17 ± 8.36

99.00 ± 8.51**

79.52 ± 5.54

76.48 ± 9.01

82.25 ± 7.58

82.86 ± 7.94

NS

NS

95.18 ± 9.70

87.76 ± 7.42*

5.55 ± 0.51

5.22 ± 0.42*

5.43 ± 0.60

5.36 ± 0.49

6.00 ± 0.27

6.45 ± 0.57**

NS

NS

6.59 ± 0.51

6.52 ± 0.51

6.00 ± 0.60

6.61

± 0.50***

4.90 ± 0.44

5.04 ± 0.35

4.93 ± 0.38

4.62 ± 0.49*

NS

NS

5.00 ± 0.00

5.10 ± 0.44

12.31 ± 0.47

12.50 ± 0.52*

10.57 ± 0.83

10.06 ± 0.72***

12.59 ± 0.47

12.76 ± 0.72

12.65 ± 0.73

11.02 ± 0.56***

11.14 ± 0.57

12.68 ± 0.73***

7.23 ± 0.45

6.937 ± 0.46*

9.19 ± 0.50

8.76

± 0.56***

8.11 ± 0.39

7.78 ± 0.52**

8.63 ± 0.50

8.16 ± 0.68**

7.45 ± 0.49

7.97

± 0.59***

4.25 ± 0.45

4.08 ± 0.43

6.35 ± 0.58

6.65 ± 0.72*

5.02 ± 0.34

5.31

± 0.52***

5.20 ± 0.58

4.75

± 0.34***

4.33 ± 0.39

5.35

± 0.61***

28.62 ± 0.60

24.22 ± 0.40***

36.64 ± 0.65

33.38 ± 0.70***

32.86 ± 0.76

36.14 ± 0.26***

32.47 ± 0.24

29.12 ± 0.61***

34.66 ±0.59

30.37 ± 0.26***

**Note:** Data are presented as mean ± SEM. Significant differences between *ZmWx1*-edited and its isogenic WT are denoted by asterisks (****p*< 0.001; ***p* < 0.01; **p* < 0.05, The two-tailed Student's t-test). NS, not scored.

**Table S4** Comparison of the agronomic traits between *ZmWx1*-edited hybrid and its wild type in the same field trial.

**Plant** **Ear Hybrids** **height Height**

**(cm)** **(cm)**

**Leaf numbers**

**Above** **Below** **Length ear ear (mm)**

**Kenerl phenotypes**

**Width** **Thickness (mm) (mm)**

**Hundred-grain weight (g)**

335 295.26 (n=42) ± 8.31

335*Wx* 295.75 (n=12) ± 7.25

106.95 6.17 5.17 ± 7.69 ± 0.38 ± 0.38

104.75 6.42 5.17 ± 12.56 ± 0.51 ± 0.39

13.48 8.76 ± 1.03 ± 0.57

14.12 8.64 ± 0.67*** ± 0.50

5.17 ± 0.93

4.62

± 0.42***

42.00 ± 0.84

40.56 ± 0.64***

**Note:** Data are presented as mean ± SEM. Significant differences between *ZmWx1*-edited and its isogenic WT are denoted by asterisks (****p*< 0.001; ***p* < 0.01; **p* < 0.05, The two-tailed Student's t-test ).

**Table S5** Top 20 genome-wide off-target of *ZmWx1* and *ZmSh2* sgRNA.

**Target** **Sequence** **Off-score MMs** **Locus** **Gene** **Region**

*ZmWx1* G**G**GGT**C**CAGCTCCG**A**GTAGT**CGG**

GA**A**GT**C**C**GC**CTCCGGGTAGT**CGG**

GAGGT**C**CA**T**C**A**CCGGGTA**T**T**CGG**

**C**AGGT**A**C**G**GCT**G**CGGGTAGT**CGG**

G**T**GGTTC**G**G**T**TCCGGGT**G**GT**CGG**

G**G**GGTTCAGC**C**CCGGG**A**AG**ACGG**

0.684 3MMs

0.269 4MMs

0.242 4MMs

0.202 4MMs

0.201 4MMs

0.16 4MMs

1:-251503590

8:-77271600

6:-135892485

2:+33978330

7:-48990146

9:-119400397

GRMZM5G805732

GRMZM2G129291

GRMZM2G127064

GRMZM2G112530

CDS

CDS

CDS

CDS

Intergenic

Intergenic

GA**A**GTTC**GC**CT**T**CGGGTAGT**CGG** 0.159 4MMs 10:-135715547 GRMZM2G387410 exon

G**CT**GTTC**G**GCT**T**CGGGTAGT**CGG**

G**CT**GTTC**G**GCT**T**CGGGTAGT**CGG**

G**CT**GTTC**G**GCT**T**CGGGTAGT**CGG**

0.155 4MMs

0.155 4MMs

0.155 4MMs

2:+48407125

7:-135139662

7:-98528877

Intergenic

Intergenic

Intergenic

GA**C**G**CA**CAGCTCCGGGTA**C**T**TGG**

**A**AGGTTCA**C**C**G**CCGGGTA**A**T**CGG**

0.149 4MMs 1:+295804457

0.138 4MMs 1:+209585798

GRMZM2G369432 CDS

Intergenic

GA**C**GT**A**CAGCTCCGGG**A**AG**ATGG**

GA**T**GT**C**CA**CG**TCCGGGTAGT**AGG**

G**C**G**C**TTCAGCT**T**CGGGT**G**GT**CGG**

GAGGTTC**G**G**GCG**CGGGTAGT**GGG**

G**G**GGT**G**C**T**GCTCCGGGT**G**GT**GGG**

**C**AGG**G**TCAGCTCC**A**GGT**C**GT**CGG**

0.1 4MMs

0.095 4MMs

0.09 4MMs

0.084 4MMs

0.078 4MMs

0.065 4MMs

5:-15789140

8:-158637852

3:-122239320

7:+23484254

2:-134972238

3:+5296478

GRMZM2G064852

GRMZM2G061403

GRMZM2G473860

CDS

CDS

Intergenic

Intergenic

exon

Intergenic

GAGGT**AG**A**T**CTCC**T**GGTAGT**GGG**

G**T**GGTTC**T**G**A**TCCGGGT**C**GT**GGG**

0.058 4MMs 2:+141223934

0.051 4MMs 4:+189548938

Intergenic

GRMZM2G428035 CDS

*ZmSh2* C**A**TGC**TA**TGGACAC**A**AACTC**AGG**

CTTGCATT**CT**A**T**AC**A**AACTC**TGG**

CTTGC**GGA**GGAC**G**CGAACTC**TGG**

CTTGCA**AA**GGA**GG**CGAACTC**CGG**

C**A**TGC**T**TTGGACAC**A**AACTC**AAG**

CTTGCA**GA**GGA**GG**CGAACTC**CGG**

0.498 4MMs

0.222 4MMs

0.219 4MMs

0.203 4MMs

0.147 3MMs

0.136 4MMs

1:-22624634

1:-256911510

5:+16645239

1:+49092744

2:-73147223

7:-77953682

GRMZM2G355358

AC207239.3_FG001

utr

Intergenic

Intergenic

Intergenic

CDS

Intergenic

**A**TTGCATTGGA**A**ACGAA**TA**C**CGG** 0.131 4MMs 6:+167902654 Intergenic

C**A**TGC**TA**TGGACAC**A**AACTC**AAG**

C**A**TGC**TA**TGGACAC**A**AACTC**AAG**

C**A**TGC**TA**TGGACAC**A**AACTC**AAG**

C**A**TGC**TA**TGGACAC**A**AACTC**AAG**

0.129 4MMs

0.129 4MMs

0.129 4MMs

0.129 4MMs

2:-130332760

6:-149788360

2:-194423233

5:+5337642

GRMZM2G000829

AC191363.3_FG006

AC209666.3_FG012

Intergenic

utr

exon

exon

C**A**TGC**TA**TGGACAC**A**AACTC**AAG**

C**A**TGC**TA**TGGACAC**A**AACTC**AAG**

C**A**TGC**TA**TGGACAC**A**AACTC**AAG**

C**A**TGC**TA**TGGACAC**A**AACTC**AAG**

0.129 4MMs 10:+69030240

0.129 4MMs 6:+128109440

0.129 4MMs 2:+209227086

0.129 4MMs 2:+220724058

GRMZM2G364779

GRMZM2G307610

exon

Intergenic

CDS

Intergenic

C**A**TGC**TA**TGGACAC**A**AACTC**AAG**

C**A**TGC**TA**TGGACAC**A**AACTC**AAG**

0.129 4MMs

0.129 4MMs

3:+29887117

3:+90435699

Intergenic

GRMZM2G063279 CDS

C**A**TGC**TA**TGGACAC**A**AACTC**AAG**

C**A**TGC**TA**TGGACAC**A**AACTC**AAG**

C**A**TGC**TA**TGGACAC**A**AACTC**AAG**

0.129 4MMs 2:+180744770

0.129 4MMs 1:+237786744

0.129 4MMs 1:+192900239

Intergenic

GRMZM2G426023 utr

Intergenic

**Table S6** The primers and probes used in this study.

**Name**

Mtl-F

Mtl-R

Dmp-F

Dmp-R

Bar-F

Bar-R

Probe-Bar

ADH1-F

ADH1-R

Probe-ADH1

Cas9-F

Cas9-R

Probe-Cas9

Wx1-F

Wx1-R

Sh2-F

Sh2-R

**Primer sequences 5ʹ–3ʹ**

cggtggctccgcaacaac

ttgggttgatggcagagacg

aacgtcgcgaaaacagttcc

caaacaaccgatgccgtagc

ctcgagtcaaatctcggtgac

gtttctggcagctggacttc

aggaccggacggggcggta

gaatgtgtgttgggtttgcat

tccagcaatccttgcacctt

tgcagcctaaccatgcgcagggta

tatcgaagttggtcatccgc

accagaaagagcgaggaaac

aagcgcccttgtccaccacttcctcgaa

agcctcaacaacaacccatactt

gagatgagctcctcggcgtag

aggtatatgcctagttccatcaaaag

gcagtgaaaccacagttcccag

**Amplicon (bp)**

376

654

74

71

103

710

681

**Usage**

*ZmMTL* mutant / WT allele identification

*ZmDMP* mutant / WT allele identification

Primers and probes for ddPCR

*ZmWx1* mutant / WT allele identification

*ZmSh2* mutant / WT allele identification
